# Golden Berry-Derived 4β-hydroxywithanolide E for Selectively Killing Oral Cancer Cells by Generating ROS, DNA Damage, and Apoptotic Pathways

**DOI:** 10.1371/journal.pone.0064739

**Published:** 2013-05-21

**Authors:** Chien-Chih Chiu, Jo-Wen Haung, Fang-Rong Chang, Kuang-Jing Huang, Hsuan-Min Huang, Hurng-Wern Huang, Chon-Kit Chou, Yang-Chang Wu, Hsueh-Wei Chang

**Affiliations:** 1 Department of Biotechnology, Kaohsiung Medical University, Kaohsiung, Taiwan; 2 Department of Biomedical Science and Environmental Biology, Kaohsiung Medical University, Kaohsiung, Taiwan; 3 Graduate Institute of Natural Products, College of Pharmacy, Kaohsiung Medical University, Kaohsiung, Taiwan; 4 Institute of Biomedical Science, National Sun Yat-Sen University, Kaohsiung, Taiwan; 5 Department of Chinese Medicine, College of Chinese Medicine, China Medical University, Taichung, Taiwan; 6 Natural Medicinal Products Research Center and Center of Molecular Cancer, China Medical University Hospital, Taichung, Taiwan; 7 Cancer Center, Kaohsiung Medical University Hospital, Kaohsiung Medical University, Kaohsiung, Taiwan; University of Windsor, Canada

## Abstract

**Background:**

Most chemotherapeutic drugs for killing cancer cells are highly cytotoxic in normal cells, which limits their clinical applications. Therefore, a continuing challenge is identifying a drug that is hypersensitive to cancer cells but has minimal deleterious effects on healthy cells. The aims of this study were to evaluate the potential of 4β-hydroxywithanolide (4βHWE) for selectively killing cancer cells and to elucidate its related mechanisms.

**Methodology and Principal Findings:**

Changes in survival, oxidative stress, DNA damage, and apoptosis signaling were compared between 4βHWE-treated oral cancer (Ca9-22) and normal fibroblast (HGF-1) cells. At 24 h and 48 h, the numbers of Ca9-22 cells were substantially decreased, but the numbers of HGF-1 cells were only slightly decreased. Additionally, the IC_50_ values for 4βHWE in the Ca9-22 cells were 3.6 and 1.9 µg/ml at 24 and 48 h, respectively. Time-dependent abnormal increases in ROS and dose-responsive mitochondrial depolarization can be exploited by using 4βHWE in chemotherapies for selectively killing cancer cells. Dose-dependent DNA damage measured by comet-nuclear extract assay and flow cytometry-based γ-H2AX/propidium iodide (PI) analysis showed relatively severer damage in the Ca9-22 cells. At both low and high concentrations, 4βHWE preferably perturbed the cell cycle in Ca9-22 cells by increasing the subG1 population and arrest of G1 or G2/M. Selective induction of apoptosis in Ca9-22 cells was further confirmed by Annexin V/PI assay, by preferential expression of phosphorylated ataxia-telangiectasia- and Rad3-related protein (p-ATR), and by cleavage of caspase 9, caspase 3, and poly ADP-ribose polymerase (PARP).

**Conclusions/Significance:**

Together, the findings of this study, particularly the improved understanding of the selective killing mechanisms of 4βHWE, can be used to improve efficiency in killing oral cancer cells during chemoprevention and therapy.

## Introduction

Oral cancer is the sixth most common cancer worldwide [1]. Its high morbidity and mortality are partly due to its relatively poor chemotherapy outcomes [2]. Because of their high cytotoxicity in normal cells, the various anti-oral cancer drugs developed so far have limited therapeutic applications. Therefore, a continuing challenge is to develop an anti-oral cancer therapy that is safer and more effective, particularly in terms of selective killing efficiency.

Extract of *Physalis peruviana* (golden berry), which is an edible plant in the family Solanaceae, reportedly confers an anti-hepatoma effect through apoptosis [3]. Our previous work [4] found that *P. peruviana*-derived 4β-hydroxywithanolide E (4βHWE) has apoptotic and antiproliferative effects on human lung cancer cells [4]. The 4βHWE withanolides are plant-derived C(28) steroidal lactones [5] with potent anti-cancer properties [6]. Other withanolides, such as withaferin A, also reportedly induce apoptosis in many cancer types, including breast cancer [7], melanoma [8], and leukemia [9].

Accumulating evidence in recent studies indicates that selective activation of apoptosis improves the effectiveness of cancer chemotherapy [10]–[15]. For improved modulation of the apoptotic potential of cancer cells, one promising line of research is the use of withanolides that selectively kill tumor cells but have low toxicity in healthy cells [13]. However, the potential use of apoptosis-inducing withanolides such as 4βHWE [4] and withaferin A [7]–[9] for selective killing, especially in oral cancer cells, is rarely discussed.

Therefore, this study examined the potential effectiveness and related mechanisms of *P. peruviana*-derived 4βHWE used for selectively killing oral cancer cells.

## Materials and Methods

### Cell cultures and drug information

Two cell lines, Ca9-22 (human gingival carcinoma) [16]–[18] and HGF-1 (human normal gingival fibroblast) [19], were cultured in Dulbecco's Modified Eagle Medium (DMEM)-F12 medium and DMEM medium (Gibco, Grand Island, NY), respectively, supplemented with 10% fetal bovine serum, 100 U/ml penicillin, 100 µg/ml streptomycin, 0.03% glutamine, and 1 mM sodium pyruvate. All cells were kept at 37°C in a humidified atmosphere containing 5% CO_2_. The 4βHWE (C_28_H_38_O_8_; MW: 502.6) was prepared from golden berry extract as described in [4] and dissolved in dimethyl sulfoxide (DMSO) for testing.

### Assessment of growth inhibition

Cell growth was measured by (3-(4,5-dimethylthiazol-2-yl)-5-(3-carboxymethoxyphenyl)-2-(4-sulfophenyl)-2H-tetrazolium (MTS) assay as described in [18]. Briefly, cells were exposed to vehicle control (DMSO) or to 4βHWE at concentrations of 1, 2, 5 and 10 µg/ml for 24 h. The cells were then exposed to MTS solution (CellTiter 96 Aqueous One Solution, Promega, Madison, WI, USA) and allowed to incubate for 1–2 h at 37°C. The product was measured at 490 nm absorbance using a Dynex MRX Model 96 Well Plate Reader (MTX Lab Systems, Inc., Vienna, VA, USA).

### Assessment of intracellular reactive oxygen species (ROS)

Intracellular ROS was measured using 2′,7′-dichlorodihydrofluorescein diacetate (DCFH-DA) from Sigma Chemical Co. (St. Louis, MO) as previously described [17]. After 4βHWE treatment, cells were washed with PBS and then incubated with 10 µM H2DCF-DA in PBS for 30 min at 37°C in darkness. Cells were then harvested and washed with PBS. After centrifugation, cells were resuspended in PBS and analyzed with a FACSCalibur flow cytometer (Becton-Dickinson, Mansfield, MA, USA) with Win-MDI software (http://facs.scripps.edu/software.html) at excitation and emission settings of 480 and 525 nm, respectively.

### Assessment of mitochondrial membrane potential

Mitochondrial membrane potential (ΔΨ_m_; MitoMP) was measured using a MitoProbe™ DiOC_2_(3) assay kit (Invitrogen, San Diego, CA, USA) as described in [16]. The 4βHWE-treated cells were suspended in 1 ml of warm PBS at approximately 1×10^6^ cells/ml, loaded with 5 µl of 10 µM DiOC_2_(3), and incubated at 37°C in 5% CO_2_ for 20–30 min. After harvest, cells were washed, resuspended in PBS, and analyzed immediately using a flow cytometer with Win-MDI software at excitation and emission settings of 488 and 525 nm, respectively.

### Assessment of DNA damage by comet-NE assay

The nuclear extract (NE) of HGF-1 cells was used to perform comet-NE assay according to a previously described protocol [4], [20] with slight modification. Briefly, cell suspensions were mixed with equal volumes of 1.2% low-melting-point agarose, immediately loaded onto 1.2% regular agarose pre-coated slides, and then cooled with ice until solidification. The third layer with an equal volume of 1.2% low-melting agarose gel was then loaded onto the solidified second gel and again cooled with ice. After cell lysis treatment at 4°C for 2 h, the slides were processed to NE digestion with a coverslip and incubated at 37°C for 2 h in a humidified space. Denaturation of the slides in 0.3 N NaOH and 1 mM EDTA for 20 min was followed by electrophoresis. After washing, the slides were transferred to 0.4 M Tris-HCl (pH 7.5), and 40 µl propidium iodide (PI, 50 µg/ml; Sigma, St Louis, MO, USA) was added for fluorescence microscopy observation (TE2000-U; Nikon, Tokyo, Japan). In the comet assay, a freeware program (http://tritekcorp.com) was used to measure DNA damage in terms of percentage of tail DNA [21].

### Assessment of DNA damage by γ-H2AX/PI cytometry

After 4βHWE treatment, cells were fixed in 70% ethanol, washed twice in BSA-T-PBS solution (1% bovine serum albumin and 0.2% Triton X-100 in PBS; Sigma), and incubated overnight at 4°C in 100 µl of BSA-T-PBS solution containing 0.2 µg p-Histone H2A.X (Ser 139) monoclonal antibody (Santa Cruz Biotechnology, Santa Cruz, CA, USA). After washing, cells were suspended for 1 h in a 1∶100 dilution of Alexa Fluor 488-tagged secondary antibody (Jackson Laboratory, Bar Harbor, ME, USA). After another washing, the cells were resuspended in 5 µg/ml of PI for analysis with a FACSCalibur flow cytometer with Win-MDI software.

### Assessment of cell cycle distribution and sub-G1 population

The PI staining was performed as described previously [20]. Briefly, cells were treated with vehicle (DMSO only) or 0.5, 1, 2, 5, and 10 µg/ml 4βHWE for 24 and 48 h. After harvest, cells were fixed overnight with 70% ethanol. After centrifugation, the cell pellets were incubated with 10 µg/ml PI and 10 µg/ml RNase A in PBS for 15 min at room temperature in darkness. The samples were then analyzed with a FACSCalibur flow cytometer and Win-MDI software.

### Assessment of apoptosis

Apoptosis was measured by annexin/PI double staining (Pharmingen, San Diego, CA, USA) as previously described [22]. Briefly, cells were treated with vehicle or with 4βHWE at doses of 1, 2, and 5 µg/ml for 24 h. The cells were then incubated with 10 µg/ml of annexin V-fluorescein isothiocyanate and 5 µg/ml of PI and analyzed with a FACSCalibur flow cytometer (Becton-Dickinson) with Win-MDI software.

### Western blotting

Western blot assay was performed as described previously [23]. Briefly, cells were first harvested and lysed. Lysates were centrifuged, and protein concentrations were determined. The 40 µg protein lysates were separated by 10% SDS-polyacrylamide gel electrophoresis and then electrotransferred. The membranes were blocked with 5% non-fat milk. The membranes were then incubated with primary antibodies against phosphorylated ataxia-telangiectasia- and Rad3-related protein (p-ATR) (#sc-109912, Ser 428, Santa Cruz Biotech., CA, USA), cleaved caspase-9 (#9501, Cell signaling Technology, Beverly, MA, USA), cleaved caspase-3 (#IMG-144A, IMGENEX, San. Diego, CA, USA), cleaved poly ADP-ribose polymerase (PARP) (#9541, Cell Signaling Technology) and β-actin (#sc-8432, Santa Cruz Biotech.), and their corresponding secondary antibodies. The ECL™ (Amersham Piscataway, NJ, USA) chemiluminescence detection kit was then used for signal detection.

### Statistical analysis

All data were presented as means ± SD. Group differences in cell viability and cell cycle were assessed by using JMP® 9 software to perform one-way ANOVA with Tukey HSD Post Hoc Test. Levels not connected by the same lower-case letter indicated significant differences. Other data were analyzed by Student *t*-test.

## Results

### Assessment of growth inhibition

**Figure 1:**
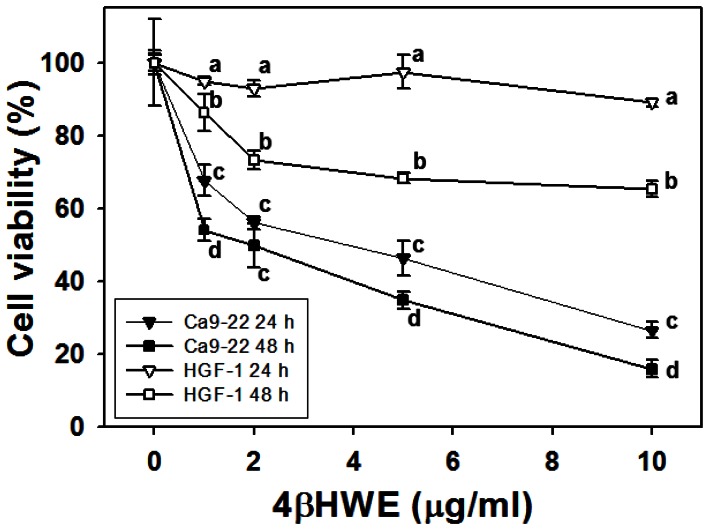
Treatment with 4βHWE induced different cell viabilities in oral cancer Ca9-22 cells and in normal oral HGF-1 cells. Cells were treated with 0, 1, 2, 5, and 10 µg/ml 4βHWE for 24 and 48 h. Data, mean±SD (n = 3). For the same drug concentrations in four different groups (Ca9-22 for 24 h, Ca9-22 for 48 h, HGF-1 for 24 h, HGF-1 for 48 h), data not connected by the same lower-case letter (upper-right corner of SD) significantly differed (one-way ANOVA with Tukey HSD Post Hoc Test).

The MTS assay of cell viability after 24 h and 48 h treatment with 4βHWE (0, 1, 2, 5 and 10 µg/ml) showed that, for each experimental concentration of 4βHWE, proliferation of Ca9-22 oral cancer cells was significantly lower than that of HGF-1 normal cells (one-way ANOVA) (Figure 1). In HGF-1 cells, treatment with 10 µg/ml 4βHWE slightly decreased cell viability to 89.02±1.17% and to 65.42±2.26% after 24 h and 48 h, respectively. In contrast, the viability of similarly treated 4βHWE-treated Ca9-22 cells dramatically decreased to 26.56±2.22 and 16.01±2.38 after 24 h and 48 h, respectively. The antiproliferative effect of 4βHWE on Ca9-22 cells was both dose-responsive and time-dependent. Additionally, IC_50_ values were 3.6 and 1.9 µg/ml after 24 h and 48 h, respectively, in 4βHWE-treated Ca9-22 cells whereas IC_50_ was undetected in similarly treated HGF-1 cells.

### Assessment of ROS

**Figure 2:**
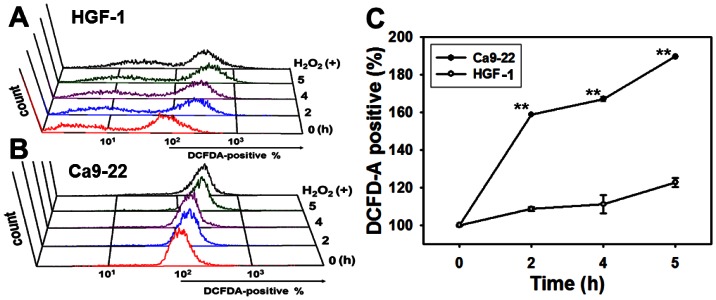
Generation of ROS differed between oral cancer Ca9-22 cells and normal oral HGF-1 cells after 4βHWE treatment. Cells were administered a vehicle and 3.6 µg/ml of 4βHWE for varying time intervals (0 to 5 h). To measure ROS change, 200 µM H_2_O_2_ was used as a positive control. (A, B) Representative flow cytometry-based ROS profiles for 4βHWE-treated cells. (C) Quantification analysis of ROS intensity in terms of DCFD-A positivity (%). The asterisks indicate significant differences between two cell lines after treatment with similar 4βHWE concentrations (*t*-test, *P*<0.0001**).

Some withanolides such as withaferin A reportedly induce cell death in melanoma cells by generating ROS [8]. Therefore, this study next compared the regulating effects of ROS on the proliferation of Ca9-22 and HGF-1 cells. Figures 2A and 2B show the ROS fluorescence intensities in terms of percentages of Ca9-22 and HGF-1 cells positive for DCFD-A, which were counted after treatment with 3.6 µg/ml 4βHWE for varying time intervals. Figure 2C shows the mild induction of ROS observed in HGF-1 cells compared to the relatively dramatic time-dependent induction of ROS in Ca9-22 cells. For each experimental concentration of 4βHWE, the percentage of DCFD-A positive cells was significantly higher in the Ca9-22 oral cancer cells than in the normal HGF-1 cells (*P*<0.0001).

### Assessment of mitochondrial membrane potentials (mitoMP)

**Figure 3:**
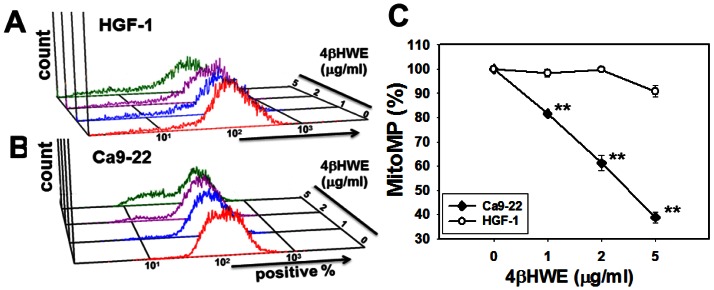
Reduction of mitochondrial membrane potential (MitoMP) differed between oral cancer Ca9-22 cells and normal oral HGF-1 cells after 4βHWE treatment. (A, B) Representative flow cytometry-based MitoMP profiles for 4βHWE-treated Ca9-22 and HGF-1 cells. Cells were treated with varying concentrations (0–5 µg/ml) of 4βHWE for 24 h. (C) Quantification analysis of MitoMP intensity. Data are presented as means ± SDs% (n = 3). The asterisks indicate statistically significant differences between the Ca9-22 and HGF-1 cell lines after treatment with similar concentrations of 4βHWE (*t*-test, *P*<0.0005**).

Next, a DiOC_2_(3) assay was performed to examine the effects of 4βHWE-induced ROS induction on mitoMP. Figures 3A and 3B show the mitoMP fluorescence intensities in terms of percentages of DiOC_2_(3)-positive Ca9-22 and HGF-1 cells after 24 h treatment with 0, 1, 2, and 5 µg/ml 4βHWE. Figure 3C shows that mitoMP moderately decreased in HGF-1 cells but substantially decreased in Ca9-22 cells in a dose-dependent manner. For each experimental concentration of 4βHWE, the percentage of cells positive for DiOC_2_(3) was significantly lower in the Ca9-22 cells than in the HGF-1 cells (*P*<0.0005).

### Assessment of DNA damage by comet-NE assay

**Figure 4:**
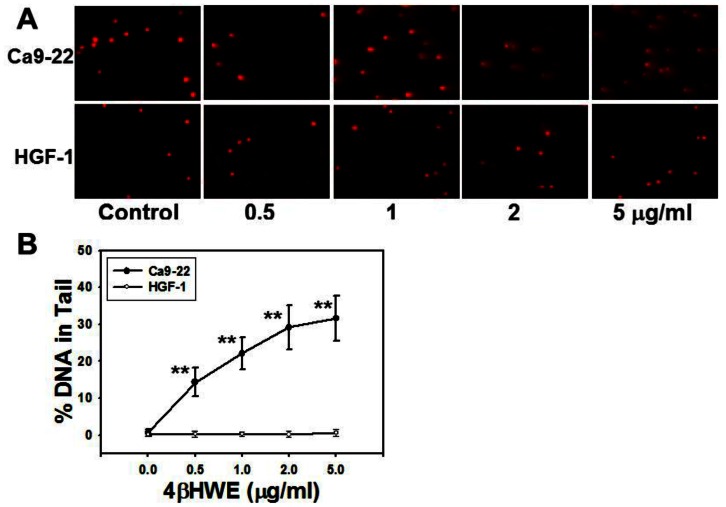
After 4βHWE treatment, oral cancer Ca9-22 cells and normal oral HGF-1 cells showed different comet-NE based DNA damage profiles. (A) Representative comet PI staining results for cell controls (DMSO) and cells treated with 0.5, 1, 2, and 5 µg/ml 4βHWE for 2 h. The nuclei appear as red spots, and the tails of the spots indicate the severity of DNA damage after 4βHWE treatments. (B) Average of the% of tail DNA in 4βHWE-treated Ca9-22 and HGF-1 cells. Data, mean±SD (nuclei = 50). Asterisks indicate significant differences between the Ca9-22 and HGF-1 cell lines after treatment with similar 4βHWE concentrations (*t*-test, *P*<0.0001**).

In the comet-NE assay (Figure 4A), the “tailing” effects observed in the Ca9-22 cells were largest at high 4βHWE concentrations. In contrast, none of the experimental 4βHWE concentrations induced an observable tailing effect in HGF-1 cells. Figure 4B shows that the HGF-1 cells showed no visible increase in% tail DNA whereas Ca9-22 cells showed dramatically increased% tail DNA in a dose-response manner. For each experimental concentration of 4βHWE, the DNA damage in terms of% DNA in the tails of cells treated with 4βHWE was significantly more severe in HGF-1 cells than in Ca9-22 cells (*P*<0.0001).

### Assessment of DNA damage by γ-H2AX/PI cytometry

**Figure 5:**
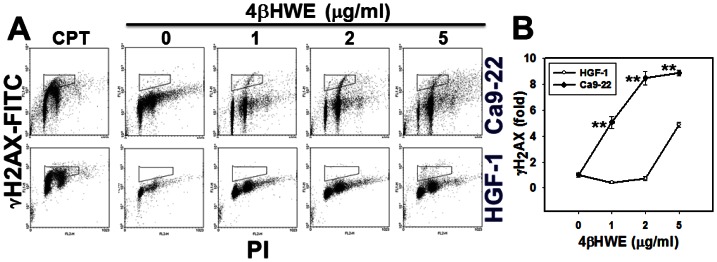
Treatment with 4βHWE induced different γ-H2AX-based DNA damage in oral cancer Ca9-22 cells and in normal oral HGF-1 cells. Cells were treated with 0, 1, 2, and 5 µg/ml of 4βHWE for 24 h. (A) Representative flow cytometry-based DSB profile for 4βHWE-treated Ca9-22 and HGF-1 cells. Cells treated with 5 µM camptothecin (CPT) for 24 h were considered γ-H2AX positive control. (B) Quantification analysis of fold changes in γ-H2AX-based DNA damage in 4βHWE-treated Ca9-22 and HGF-1 cells. Data, mean±SD. The asterisks indicate statistically significant differences between Ca9-22 and HGF-1 cells treated with similar 4βHWE concentrations (*t*-test, *P*<0.0005**; n = 2 and 3, respectively).

For further confirmation of the involvement of DNA damage in 4βHWE-induced inhibition of growth in Ca9-22 oral cancer cells, the DNA double strand break (DSB) was measured in terms of γ-H2AX expression. Figure 5A displays the γ-H2AX/PI staining profiles obtained after 24 h treatments with positive control or with 0, 0.1, 0.25, 0.5 and 1 µg/ml of 4βHWE. Figure 5B shows that, after treatment with 4βHWE concentrations lower than 2 µg/ml, the HGF-1 cells maintained low levels of γ-H2AX expression whereas Ca9-22 cells showed dramatic dose-dependent increases in γ-H2AX expression. At higher concentrations of 4βHWE, however, the fold change in the% of γ-H2AX-positive cells was significantly larger in Ca9-22 cells than in HGF-1 cells (*P*<0.0005).

### Assessment of cell cycle distribution

**Figure 6:**
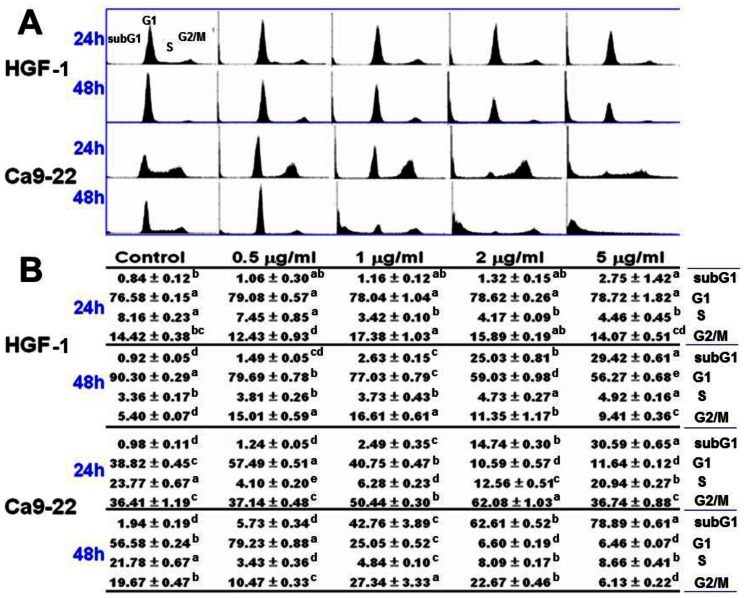
Treatment with 4βHWE induced different accumulations of subG1 in oral cancer Ca9-22 cells and in normal oral HGF-1 cells. Cells were treated with 0, 1, 2, and 5 µg/ml 4βHWE for 24 h and 48 h. (A) Representative cell cycle distribution in 4βHWE-treated Ca9-22 and HGF-1 cells. (B) Cell phase percentages obtained for (A) in triplicate experiments. For the same phase of different drug concentrations, data not connected by the same lower-case letter (upper right of SD) significantly differed (one-way ANOVA with Tukey HSD Post Hoc Test).

Figure 6 shows that, after 24 h treatment with 4βHWE, the percentage change in sub-G1 populations in HGF-1 cells was not statistically significant at concentrations lower than 2 µg/ml. The percentage change in sub-G1 slightly increased to 2.75% when the concentration reached 5 µg/ml. In contrast, the percentage change in sub-G1 in Ca9-22 cells treated with 4βHWE for 24 h began showing significant changes at concentrations as low as 2 µg/ml and eventually reached 30.59%. After 48 h treatment, the percentage of sub-G1 populations in HGF-1 cells moderately increased to 25.03% and 29.42% after treatments with 2 and 5 µg/ml 4βHWE, respectively. In contrast, the percentage of sub-G1 in Ca9-22 cells treated with 4βHWE for 48 h showed a statistically significant change (42.76%) after treatment with 1 µg/ml and a much larger change (78.89%) after treatment with 5 µg/ml.

At 24 h, analyses of G1 and G2/M populations in HGF-1 cells showed a non-significant change in the G1 population and a basal level of change in the G2/M pollution. In contrast, after 24 h 4βHWE treatment, the Ca9-22 cells showed significant changes in G1 arrest (57.49% and 40.75% at 0.5 and 1 µg/ml, respectively) and in G2/M arrest (50.44% and 62.08% at 1 and 2 µg/ml, respectively). After 48 h treatment with 5 µg/ml βHWE, the G1 population in the HGF-1 cells slightly decreased to 56.27% whereas the G2/M population slightly increased. In contrast, the Ca9-22 cells showed substantial (79.23%) G1 arrest after 48 h treatment with a 4βHWE concentration of only 0.5 µg/ml. In the G2/M population, treatment with 1 µg/ml 4βHWE resulted in moderate (27.34%) G1 arrest coupled with dramatic subG1 accumulations as described above.

### Assessment of apoptosis

**Figure 7:**
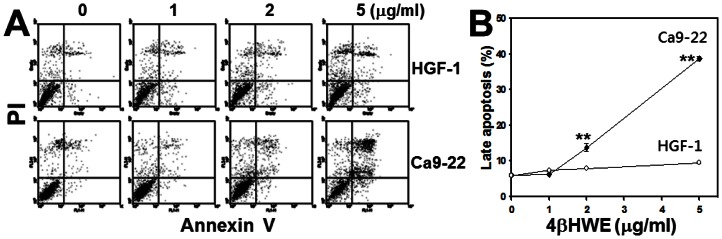
Treatment with 4βHWE induced different apoptotic profiles in oral cancer Ca9-22 cells and in normal oral HGF-1 cells. Cells were treated with 0, 1, 2, and 5 µg/ml 4βHWE for 24 h. (A) Representative apoptotic profiles obtained by Annexin V/PI double staining in 4βHWE-treated Ca9-22 and HGF-1 cells. (B) Quantification analysis results for late apoptosis population (%). Only annexin V (+)/PI (+) regions were analyzed. Data, mean±SD (n = 3). Asterisks indicate statistically significant differences between two cell lines (Ca9-22 and HGF-1) treated with similar 4βHWE concentrations (*t*-test, *P*<0.0001**).

To examine the involvement of apoptosis in 4βHWE-induced sub-G1 accumulation, flow cytometry-based annexin V/PI double staining was performed. Figure 7A shows the γ-H2AX/PI staining profiles of HGF-1 and Ca9-22 cells treated with varying 4βHWE concentrations. The double positive areas of γ-H2AX and PI intensities are commonly defined as late apoptosis. Analyses of late apoptosis (Figure 7B) showed a mild dose-dependent increase in HGF-1 cells but a dramatic dose-dependent increase in Ca9-22 cells. For each experimental concentration of 4βHWE, the percentage of cells that underwent late apoptosis after 4βHWE treatment was significantly higher in Ca9-22 cells than in HGF-1 cells (*P*<0.0001).

### Assessment of apoptotic signaling

**Figure 8:**
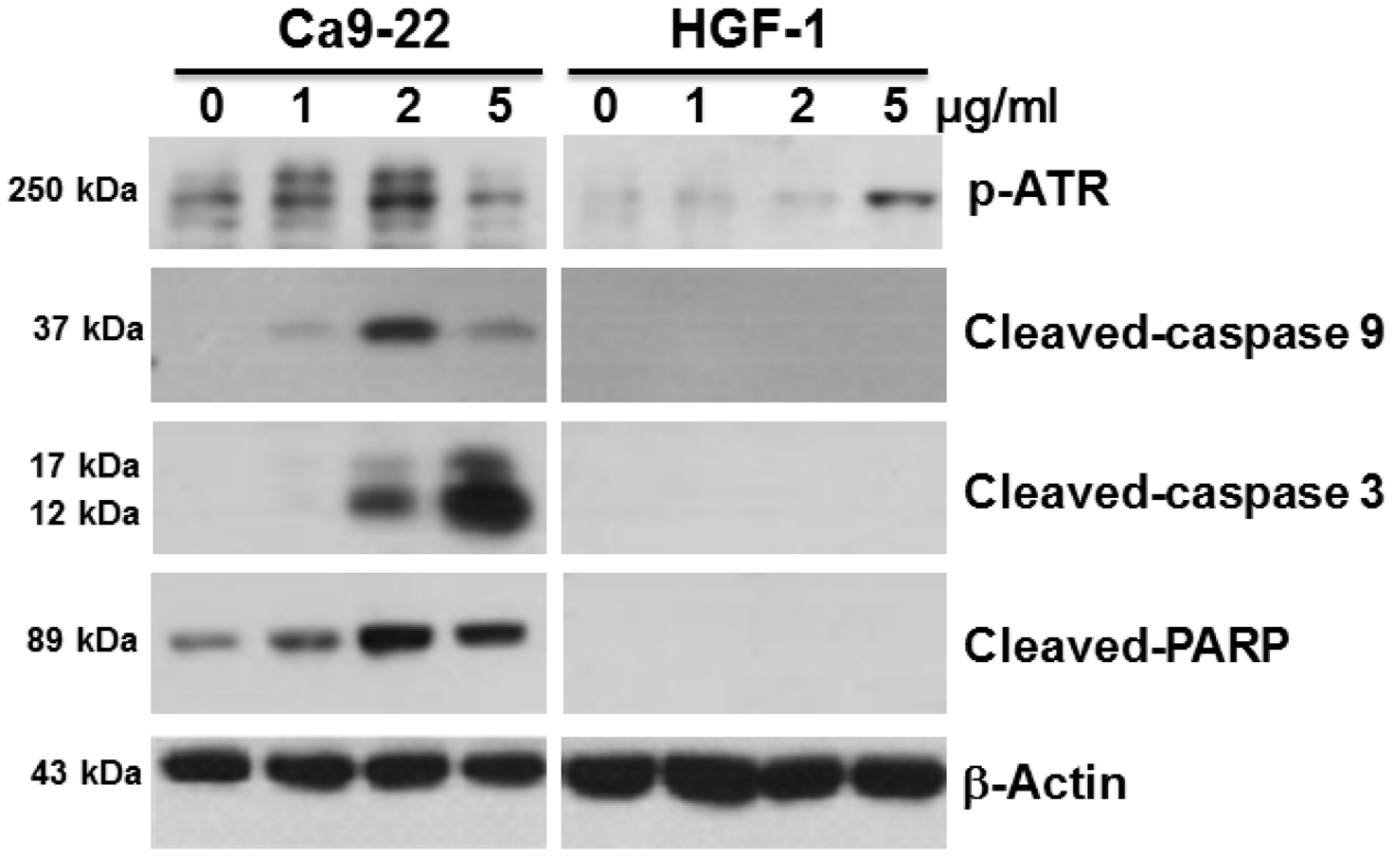
Treatment with 4βHWE induced different apoptosis-related protein expressions in oral cancer Ca9-22 cells and in normal oral HGF-1 cells. Cells were treated with 0, 1, 2, and 5 µg/ml 4βHWE for 24 h. Apoptosis signaling proteins, such as p-ATR, and cleavage of pro-caspase 9, pro-caspase 3 and PARP were detected. The β-actin was used as an internal control. Each blot represents an independent experiment performed in triplicate.

Apoptotic signaling was examined by Western blotting assays of Ca9-22 and HGF-1 cells treated with varying concentrations of 4βHWE (0, 1, 2, and 5 µg/ml). Figure 8 shows that increasing concentrations of 4βHWE induced stepwise increases in ATR phosphorylation associated with cellular DNA damage in the Ca9-22 cells. Cleavage of caspase 9, caspase 3 and PARP was also induced in Ca9-22 cells, especially at 4βHWE concentrations of 2 µg/ml and 5 µg/ml. In contrast, 4βHWE did not trigger either ATR phosphorylation or activation of caspase cascade in the HGF-1 cells.

## Discussion

Withanolides are plant-derived C(28) steroidal lactones with potent anti-cancer activity [24], [25]. However, the use of withanolides for selectively killing cancer cells has not been intensively studied. Withaferin A is known to induce apoptosis in many cancer types, including leukemia [9], melanoma [8], and cancers of the breast [7], liver [26], pancreas [27], colon [28], lung [29], and prostate [30]. In earlier studies, XTT assays performed after 24 h treatment with withaferin A showed IC_50_ values of 2 µM in leukemia cells (HL-60) [31], 4 µM in prostate cancer cells (PC3) [30], and>6 µM in hepatoma (SK-Hep1), colon cancer (HT29), and renal cancer (Caki) cells [26]. Reported IC_50_ values obtained by MTT assays of breast cancer cells (MDA-MB-231) include 13 µM for anomanolide A, 15 µM for tubocapsanolide E, and>20 µM for tubocapsenolide B, tubocapsanolide C, and peruvianolide H [6].

Recently, withanolide derived from Ashwagandha leaf extract [32] has shown a potential role in selectively killing breast cancer MCF7 cells at a concentration of 24 µg/ml [33]. The current study similarly showed that the IC_50_ values of Ca9-22 oral cancer cells were 3.6 µg/ml (7.16 µM) and 1.9 µg/ml (3.78 µM) after 24 h and 48 h treatment with 4βHWE, respectively. We hypothesize that the selective killing effect of 4βHWE may be comparable or even more potent in other oral cancer cell lines. In HGF-1 cells, however, IC_50_ values were undetectable by MTS assay at concentrations lower than 10 µg/ml. In other studies of 4βHWE treatments for 24 h, an IC_50_ value of 6 µM was reported in an MTT assay of breast cancer MDA-MB-231 cells [6] and an IC_50_ value of 1.41 µM was reported in a trypan blue assay of lung cancer H1299 cells [4]. These data indicate that the drug sensitivity of 4βHWE varies according to cancer cell type. Moreover, the current study is the first to confirm that 4βHWE kills oral cancer cells preferentially to normal oral cells.

Furthermore, the protective effects of withanolides are found in normal fibroblast cells. For example, withanone derived from the Ashwagandha leaf protects normal human fibroblasts against methoxyacetic acid-induced senescence-like growth arrest in terms of senescence-associated β-galactosidase staining [34]. Similarly, the current study found that the morphology of HGF-1 cells treated with 1, 2, and 5 µg/ml 4βHWE was similar to that of untreated fibroblast HGF-1 cells (data not shown). Further studies of growth arrest phenotypes are needed to determine whether 4βHWE is safe for normal cells.

Although accumulating evidence agrees that withanolides induce ROS-mediated apoptosis, their potential use for selectively killing cancer cells is relatively unclear. For example, withaferin A is known to induce ROS-mediated apoptosis in breast cancer [7], melanoma [8], and leukemia [9], [31]. Although cancer cells are expected to have higher oxidative stress compared to normal cells [35], normal cells may tolerate an exogenous oxidative stress level sufficient to prevent overload resulting in cell death. In contrast, cancer cells exposed to high oxidative stress cannot tolerate exogenous ROS-modulating agents, and cell death increases as the threshold level is exceeded [36]. This concept may partly explain the selective killing effects of 4βHWE in oral cancer cells observed here and those of withanone in breast cancer cells as reported in [33], i.e., both ROS induction and the reduction of mitochondrial membrane potential are higher in cancer cells compared to normal cells. These results suggest that therapies for selectively killing cancer cells should be targeted at modulating redox status in both cancer cells and normal cells [36]. However, the roles of caspases and caspase inhibitors [37] in 4βHWE-induced selective apoptosis need further study.

The ROS-mediated effects of withanolides may be modulated by antioxidants. For example, N-acetylcysteine can reportedly rescue human melanoma cells from withaferin A-induced, ROS-mediated apoptosis [8]. Accordingly, the role of ROS in the selective killing of 4βHWE may be further clarified by studying ROS modulators.

The ROS are known to induce DNA damage and checkpoint responses [38]. For example, the comet-NE and γ-H2AX assays in this study revealed that 4βHWE induced DNA damage and G1 or G2/M cell cycle arrest, both of which have been observed earlier in other withanolides. For example, tubocapsanolide A reportedly inhibits proliferation of lung cancer A549 cells via G1 arrest [39]. However, 4βHWE [4] and withanone [33] are known to induce DNA damage and then arrest at G2/M in lung cancer H1299 cells and in breast cancer MCF-7 cells, respectively.

Selective DNA damage (i.e., DSB) can be monitored by H2AX, which is phosphorylated in an ATR-dependent manner [40]. The H2AX also helps to stabilize the genome [41] and is essential for caspase-activated DNA fragmentation [42]. As expected, 4βHWE treatment induced higher expressions of ATR and caspase signaling proteins in Ca9-22 cells compared to HGF-1 cells.

After 24 h treatment with 2 µg/ml 4βHWE, Ca9-22 and HGF-1 cells showed cell viabilities (%) of 56.09±1.78 and 92.81±2.20 (Figure 1); mitoMP (%) of 61.18±3.21 and 99.81±0.74 (Figure 3); γ-H2AX (fold) of 8.47±0.54 and 0.73±0.19 (Figure 5); subG1 populations (%) of 14.74±0.30 and 1.32±0.15; G2/M arrests of 62.08±1.03 and 15.89±0.19 (Figure 6) late apoptosis (%) of 13.80±1.05 and 7.97±0.06 (Figure 7); and over- and under-expression of apoptosis signaling proteins (Figure 8), respectively. These results consistently showed the effects of 4βHWE in terms of selective killing, selective mitochondrial dysfunction, selective DNA damage (i.e., DSB), selective G2/M arrest, and selective apoptosis of Ca9-22 cells preferentially to HGF-1 cells. After similar treatment with 3.6 µg/ml 4βHWE for 2 h, the Ca9-22 and HGF-1 cells showed ROS induction (%) of 158.83±0.34 and 108.63±1.04 (Figure 2) and comet-NE-based DNA damage of 29.21±5.93 and 0.27±0.52 (Figure 4), respectively. These results further confirmed that 4βHWE treatment selectively induces ROS and DNA damage in Ca9-22 cells in preference to HGF-1 cells.

**Figure 9:**
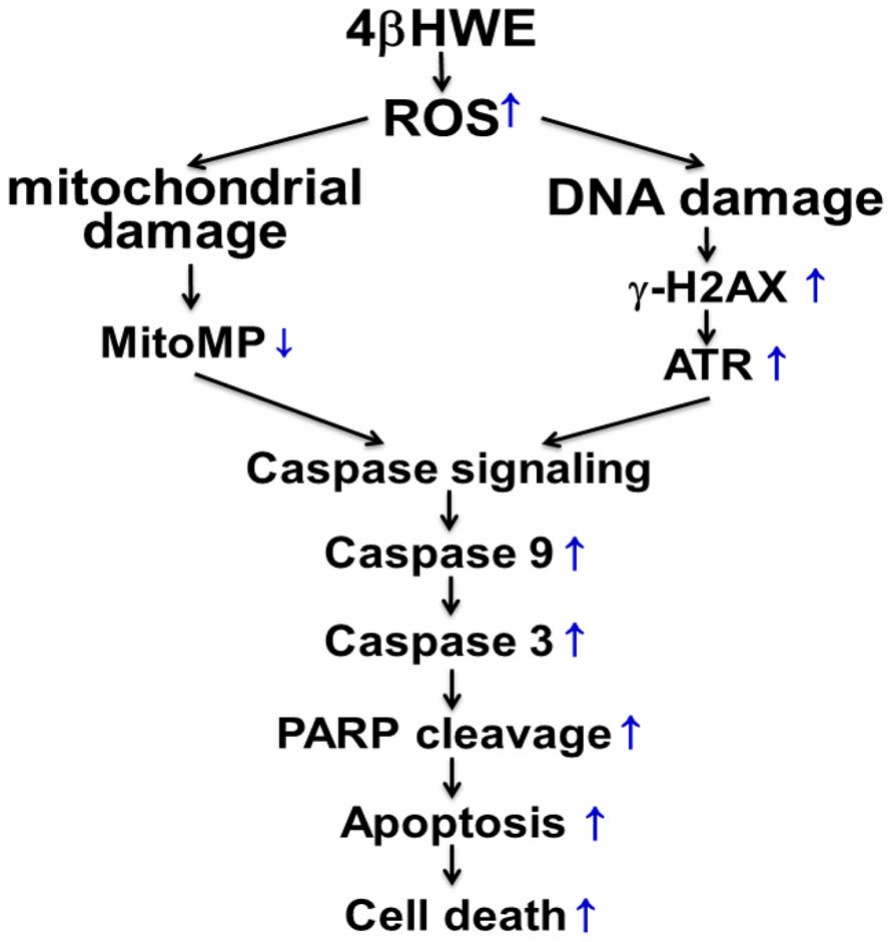
Schematic diagram of hypothesized mechanism of 4βHWE-induced selective killing of human oral cancer Ca9-22 cells preferentially to normal oral HGF-1 cells. Changes in Ca9-22 cells are indicated by blue arrows. Normal oral cells (not shown) showed relatively smaller changes compared to Ca9-22 cells. In Ca9-22 treated with 4βHWE, enhanced ROS generation resulted in increased oxidative stress. The induction of ROS then increased mitochondrial dysfunction and facilitated reduction of mitochondrial membrane potential in Ca9-22 cells. Induction of ROS also resulted in increased DNA damage in Ca9-22 cells, e.g., γ-H2AX in DSB, which then induced ATR phosphorylation. Together, mitochondrial damage signaling and DNA damage signaling resulted in increased apoptosis signaling, which then led to selective apoptosis and killing of Ca9-22 cells.

In conclusion, the results of this study confirm that 4βHWE treatment selectively induces ROS, mitochondrial depolarization, and DNA damage, which in turn induces selective apoptosis signaling, which ultimately results in the selective killing of oral cancer cells (Figure 9). Accordingly, this study showed, for the first time, that 4βHWE treatment selectively kills oral cancer cells in preference to normal oral cells. Further study of the target candidates and signaling mechanisms reported here may also provide a sufficiently improved understanding of the selective killing mechanisms of 4βHWE to enable its effective use in treating oral cancer with minimal adverse effects.

## References

[1] Parkin DM, Laara E, Muir CS (1988) Estimates of the worldwide frequency of sixteen major cancers in 1980. Int J Cancer 41: 184–197. 3338870 10.1002/ijc.2910410205

[2] Myoung H, Hong SP, Yun PY, Lee JH, Kim MJ (2003) Anti-cancer effect of genistein in oral squamous cell carcinoma with respect to angiogenesis and in vitro invasion. Cancer Sci 94: 215–220. 12708500 10.1111/j.1349-7006.2003.tb01422.x PMC11160255

[3] Wu SJ, Ng LT, Chen CH, Lin DL, Wang SS, et al (2004) Antihepatoma activity of *Physalis angulata* and *P. peruviana* extracts and their effects on apoptosis in human Hep G2 cells. Life Sci 74: 2061–2073. 14967200 10.1016/j.lfs.2003.09.058

[4] Yen CY, Chiu CC, Chang FR, Chen JY, Hwang CC, et al (2010) 4beta-Hydroxywithanolide E from *Physalis peruviana* (golden berry) inhibits growth of human lung cancer cells through DNA damage, apoptosis and G2/M arrest. BMC Cancer 10: 46. 20167063 10.1186/1471-2407-10-46 PMC2830937

[5] Vaishnavi K, Saxena N, Shah N, Singh R, Manjunath K, et al (2012) Differential activities of the two closely related withanolides, Withaferin A and Withanone: bioinformatics and experimental evidences. PLoS One 7: e44419. 22973447 10.1371/journal.pone.0044419 PMC3433425

[6] Wang HC, Tsai YL, Wu YC, Chang FR, Liu MH, et al (2012) Withanolides-induced breast cancer cell death is correlated with their ability to inhibit heat protein 90. PLoS One 7: e37764. 22701533 10.1371/journal.pone.0037764 PMC3365124

[7] Hahm ER, Moura MB, Kelley EE, Van Houten B, Shiva S, et al (2011) Withaferin A-induced apoptosis in human breast cancer cells is mediated by reactive oxygen species. PLoS One 6: e23354. 21853114 10.1371/journal.pone.0023354 PMC3154436

[8] Mayola E, Gallerne C, Esposti DD, Martel C, Pervaiz S, et al (2011) Withaferin A induces apoptosis in human melanoma cells through generation of reactive oxygen species and down-regulation of Bcl-2. Apoptosis 16: 1014–1027. 21710254 10.1007/s10495-011-0625-x

[9] Mandal C, Dutta A, Mallick A, Chandra S, Misra L, et al (2008) Withaferin A induces apoptosis by activating p38 mitogen-activated protein kinase signaling cascade in leukemic cells of lymphoid and myeloid origin through mitochondrial death cascade. Apoptosis 13: 1450–1464. 18987975 10.1007/s10495-008-0271-0

[10] Abdullah NM, Rosania GR, Shedden K (2009) Selective targeting of tumorigenic cancer cell lines by microtubule inhibitors. PLoS One 4: e4470. 19214225 10.1371/journal.pone.0004470 PMC2636860

[11] Lubin M, Lubin A (2009) Selective killing of tumors deficient in methylthioadenosine phosphorylase: a novel strategy. PLoS One 4: e5735. 19478948 10.1371/journal.pone.0005735 PMC2684647

[12] Daniel D, Susal C, Kopp B, Opelz G, Terness P (2003) Apoptosis-mediated selective killing of malignant cells by cardiac steroids: maintenance of cytotoxicity and loss of cardiac activity of chemically modified derivatives. Int Immunopharmacol 3: 1791–1801. 14636829 10.1016/j.intimp.2003.08.004

[13] Ovadje P, Chatterjee S, Griffin C, Tran C, Hamm C, et al (2011) Selective induction of apoptosis through activation of caspase-8 in human leukemia cells (Jurkat) by dandelion root extract. J Ethnopharmacol 133: 86–91. 20849941 10.1016/j.jep.2010.09.005

[14] Iseki S, Nakamura K, Hayashi M, Tanaka H, Kondo H, et al (2012) Selective killing of ovarian cancer cells through induction of apoptosis by nonequilibrium atmospheric pressure plasma. Appl Phys Lett 100: 113702.

[15] Akhtar MJ, Ahamed M, Kumar S, Khan MM, Ahmad J, et al (2012) Zinc oxide nanoparticles selectively induce apoptosis in human cancer cells through reactive oxygen species. Int J Nanomedicine 7: 845–857. 22393286 10.2147/IJN.S29129 PMC3289443

[16] Yen CY, Chiu CC, Haung RW, Yeh CC, Huang KJ, et al (2012) Antiproliferative effects of goniothalamin on Ca9-22 oral cancer cells through apoptosis; DNA damage and ROS induction. Mutat Res 747: 253–258. 22721813 10.1016/j.mrgentox.2012.06.003

[17] Yeh CC, Yang JI, Lee JC, Tseng CN, Chan YC, et al (2012) Anti-proliferative effect of methanolic extract of *Gracilaria tenuistipitata* on oral cancer cells involves apoptosis, DNA damage, and oxidative stress. BMC Complement Altern Med 12: 142. 22937998 10.1186/1472-6882-12-142 PMC3495219

[18] Yeh CC, Tseng CN, Yang JI, Huang HW, Fang Y, et al (2012) Antiproliferation and induction of apoptosis in Ca9-22 oral cancer cells by ethanolic extract of *Gracilaria tenuistipitata* . Molecules 17: 10916–10927. 22968475 10.3390/molecules170910916 PMC6269058

[19] Chiang SL, Jiang SS, Wang YJ, Chiang HC, Chen PH, et al (2007) Characterization of arecoline-induced effects on cytotoxicity in normal human gingival fibroblasts by global gene expression profiling. Toxicol Sci 100: 66–74. 17682004 10.1093/toxsci/kfm201

[20] Chiu CC, Chang HW, Chuang DW, Chang FR, Chang YC, et al (2009) Fern plant-derived protoapigenone leads to DNA damage, apoptosis, and G(2)/m arrest in lung cancer cell line H1299. DNA Cell Biol 28: 501–506. 19630532 10.1089/dna.2009.0852

[21] Collins AR (2004) The comet assay for DNA damage and repair: principles, applications, and limitations. Mol Biotechnol 26: 249–261. 15004294 10.1385/MB:26:3:249

[22] Chiu CC, Liu PL, Huang KJ, Wang HM, Chang KF, et al (2011) Goniothalamin inhibits growth of human lung cancer cells through DNA damage, apoptosis, and reduced migration ability. J Agric Food Chem 59: 4288–4293. 21391609 10.1021/jf200566a

[23] Chiu CC, Chen JYF, Lin KL, Huang CJ, Lee JC, et al (2010) p38 MAPK and NF-κB pathways are involved in naphtho [1, 2-b] furan-4, 5-dione induced anti-proliferation and apoptosis of human hepatoma cells. Cancer Letters 295: 92–99. 20350781 10.1016/j.canlet.2010.02.017

[24] Khodaei M, Jafari M, Noori M (2012) Remedial use of withanolides from *Withania coagolans* (Stocks) Dunal. Advances in Life Sciences 2: 6–19.

[25] Singh A, Duggal S, Singh H, Singh J, Katekhaye S (2010) Withanolides: Phytoconstituents with significant pharmacological activities. Int J Green Pharm 4: 229–237.

[26] Choi MJ, Park EJ, Min KJ, Park JW, Kwon TK (2011) Endoplasmic reticulum stress mediates withaferin A-induced apoptosis in human renal carcinoma cells. Toxicol In Vitro 25: 692–698. 21266191 10.1016/j.tiv.2011.01.010

[27] Yu Y, Hamza A, Zhang T, Gu M, Zou P, et al (2010) Withaferin A targets heat shock protein 90 in pancreatic cancer cells. Biochem Pharmacol 79: 542–551. 19769945 10.1016/j.bcp.2009.09.017 PMC2794909

[28] Koduru S, Kumar R, Srinivasan S, Evers MB, Damodaran C (2010) Notch-1 inhibition by Withaferin-A: a therapeutic target against colon carcinogenesis. Mol Cancer Ther 9: 202–210. 20053782 10.1158/1535-7163.MCT-09-0771 PMC3041017

[29] Choudhary MI, Hussain S, Yousuf S, Dar A, Mudassar, et al (2010) Chlorinated and diepoxy withanolides from *Withania somnifera* and their cytotoxic effects against human lung cancer cell line. Phytochemistry 71: 2205–2209. 21044792 10.1016/j.phytochem.2010.08.019

[30] Srinivasan S, Ranga RS, Burikhanov R, Han SS, Chendil D (2007) Par-4-dependent apoptosis by the dietary compound withaferin A in prostate cancer cells. Cancer Res 67: 246–253. 17185378 10.1158/0008-5472.CAN-06-2430

[31] Malik F, Kumar A, Bhushan S, Khan S, Bhatia A, et al (2007) Reactive oxygen species generation and mitochondrial dysfunction in the apoptotic cell death of human myeloid leukemia HL-60 cells by a dietary compound withaferin A with concomitant protection by N-acetyl cysteine. Apoptosis 12: 2115–2133. 17874299 10.1007/s10495-007-0129-x

[32] Widodo N, Kaur K, Shrestha BG, Takagi Y, Ishii T, et al (2007) Selective killing of cancer cells by leaf extract of Ashwagandha: identification of a tumor-inhibitory factor and the first molecular insights to its effect. Clin Cancer Res 13: 2298–2306. 17404115 10.1158/1078-0432.CCR-06-0948

[33] Widodo N, Priyandoko D, Shah N, Wadhwa R, Kaul SC (2010) Selective killing of cancer cells by Ashwagandha leaf extract and its component Withanone involves ROS signaling. PLoS One 5: e13536. 20975835 10.1371/journal.pone.0013536 PMC2958829

[34] Priyandoko D, Ishii T, Kaul SC, Wadhwa R (2011) Ashwagandha leaf derived withanone protects normal human cells against the toxicity of methoxyacetic acid, a major industrial metabolite. PLoS One 6: e19552. 21573189 10.1371/journal.pone.0019552 PMC3087802

[35] Nicco C, Laurent A, Chereau C, Weill B, Batteux F (2005) Differential modulation of normal and tumor cell proliferation by reactive oxygen species. Biomed Pharmacother 59: 169–174. 15862711 10.1016/j.biopha.2005.03.009

[36] Sun Y, St Clair DK, Xu Y, Crooks PA, St Clair WH (2010) A NADPH oxidase-dependent redox signaling pathway mediates the selective radiosensitization effect of parthenolide in prostate cancer cells. Cancer Res 70: 2880–2890. 20233868 10.1158/0008-5472.CAN-09-4572 PMC2848907

[37] Fricker M, Vilalta A, Tolkovsky AM, Brown GC (2013) Caspase inhibitors protect neurons by enabling selective necroptosis of inflamed microglia. J Biol Chem 288: 9145–9152. 23386613 10.1074/jbc.M112.427880 PMC3610987

[38] Guachalla LM, Rudolph KL (2010) ROS induced DNA damage and checkpoint responses: influences on aging? Cell Cycle 9: 4058–4060. 20935491 10.4161/cc.9.20.13577

[39] Chang HC, Chang FR, Wang YC, Pan MR, Hung WC, et al (2007) A bioactive withanolide Tubocapsanolide A inhibits proliferation of human lung cancer cells via repressing Skp2 expression. Mol Cancer Ther 6: 1572–1578. 17513606 10.1158/1535-7163.MCT-06-0812

[40] Ward IM, Chen J (2001) Histone H2AX is phosphorylated in an ATR-dependent manner in response to replicational stress. J Biol Chem 276: 47759–47762. 11673449 10.1074/jbc.C100569200

[41] Chanoux RA, Yin B, Urtishak KA, Asare A, Bassing CH, et al (2009) ATR and H2AX cooperate in maintaining genome stability under replication stress. J Biol Chem 284: 5994–6003. 19049966 10.1074/jbc.M806739200 PMC2645842

[42] Lu C, Zhu F, Cho YY, Tang F, Zykova T, et al (2006) Cell apoptosis: requirement of H2AX in DNA ladder formation, but not for the activation of caspase-3. Mol Cell 23: 121–132. 16818236 10.1016/j.molcel.2006.05.023 PMC2227311

